# Impact of habitat associations on saproxylic beetle assemblages and their damage severity

**DOI:** 10.1038/s41598-025-20452-5

**Published:** 2025-10-21

**Authors:** Bat-Amgalan Batchudur, Nanzaddorj Tsagaantsooj, Dashzeveg Ganbat, Bazartseren Boldgiv

**Affiliations:** 1 Mongolian Academy of Sciences, Division of Forest Resource and Forest Protection, Institute of Geoecology and Geography, Ulaanbaatar, 15170 Mongolia; 2 Department of Biology, National University of Mongolia, Ulaanbaatar, 210646 Mongolia

**Keywords:** Saproxylic beetle, Damage index, Feeding guilds, Downed tree species, Decay class, Green zone of Ulaanbaatar city, Ecology, Ecology

## Abstract

Saproxylic beetles, as primary decomposers in forest ecosystems, play a crucial role in the decomposition of dead wood. However, there is a significant gap in understanding the extent of assemblages and damage caused by these insects, which is essential for managing the quality and utilization of dead wood resources in natural forests. This study employed the Bevan damage classification system to quantify the severity of saproxylic beetle damage to fallen trees, focusing on the boreal forest in the Green zone of Ulaanbaatar, the capital of Mongolia. A factorial design was used to assess the influence of forest landscape (north vs. south mixed forest), tree species (Siberian spruce *Picea obovata* and Siberian pine *Pinus sibirica*), and decay class (1–4) on beetle damage indices, abundance and feeding guilds (cambium consumers, wood borers, predators, parasitoids, and detritivores). Our findings reveal that decay class significantly affects beetle abundance and damage severity with early stages showing the highest values. Cambium consumers and wood borers were more abundant in decay class 1 (DC1) for downed spruce, with *Ips typographus* (24.7%) and *Tetropium castaneum* (15%) causing the most damage. For the Siberian pine, *Monochamus galloprovincialis* (9.8%) and *Judolia sexmaculata* (13.3%) were the most damaging in DC1 followed by *Pityogenes conjunctus* (10%). The results suggest that Siberian spruce may be more susceptible to saproxylic beetle damage than the Siberian pine, with structural features such as bark cover branch size and wood moisture playing a critical role, especially in early decay stages. Based on our findings, we recommend decay-stage-specific management approaches, particularly targeting early decay stages (DC1–DC2) where beetle damage is most severe. Practical strategies include early detection of freshly downed trees, bark removal to reduce suitable habitat for cambium consumers, and on-site processing techniques such as bark gouging or mechanical debarking. These methods allow deadwood biomass to be retained in the forest while reducing pest pressure, offering a viable alternative to salvage logging. Such approaches are especially relevant in protected areas, where they can support both pest control and biodiversity conservation objectives. However, given the geographic scope limited to boreal forests of Ulaanbaatar, caution should be exercised in extrapolating these recommendations to other regions without further study.

**Supplementary Information:**

The online version contains supplementary material available at 10.1038/s41598-025-20452-5.

## Introduction

Saproxylic beetles—those that depend on dead or decaying wood – are essential decomposers that play a key role in nutrient cycling in forest ecosystems, yet they pose significant economic challenges by causing damage to timber resources, impacting both biodiversity and forest management strategies^1,2^. This dual role is particularly pronounced in Siberian conifer forests in Mongolia^3–5^, where commercially valuable species like Siberian spruce (*P. obovata*) and Siberian pine (*P. sibirica*) dominate^6–9^. These forests often face natural disturbances such as strong winds that can lead to uprooting, creating a substantial amount of wind-fallen dead wood. Especially, natural coniferous forests in the Green zone of Ulaanbaatar, the capital city of Mongolia, were seriously impacted by wind in the spring of 2020 and in the years before compared to other types of forest ecosystems. As a result, many Siberian pine and spruce trees have been uprooted, and the lack of utilization of these wood resources for commercial and industrial purposes is causing significant economic losses. While this dead wood serves as critical habitat for saproxylic insects, fostering biodiversity and ecosystem functions, it also presents challenges for forest management due to the potential degradation of wood quality^10,11^.

Although saproxylic beetles and their ecological roles have been studied in temperate and boreal forests across Europe and North America, little is known about their diversity, feeding guilds, and damage severity in the forest ecosystems of Mongolia^11,12^. Previous studies have focused on insects causing damage to harvested material held in outdoor storage and living trees rather than on natural dead wood in Turkey^13,14^. This study of saproxylic beetle communities in their natural fallen wood environments is the first to allow for more accurate assessment of their ecological role, economic impact and the progression of wood decomposition.

Bevan Damage Index, a pivotal metric in our study, quantifies the extent of damage inflicted by saproxylic beetles across different tree species and decay classes, providing important insights for the development of targeted management strategies. Early colonizers, such as the larvae of bark beetles and wood-boring beetles, play a critical role by initiating the decomposition process that, while crucial for forest regeneration, cause substantial physical and technical damage. This damage, characterized by extensive tunneling through the wood, not only compromises the structural integrity of the logs but also expands their surface area, thereby enhancing microbial activity and further decomposition^15–17^.

Beetles belonging to families such as Cerambycidae, Anobiidae, Bostrichidae, Buprestidae or Melandryidae can result in significant economic losses by causing damage to lumber, thereby reducing its technical qualities^18^. These insects not only target different stages and types of wood, but also display diverse feeding behaviors and survival strategies, which contribute to the complexity and severity of their infestations. Cerambycidae, in particular, are known for their broad spectrum of damage, affecting a wide range of wood species and highlighting the pervasive nature of these infestations in wood ecosystems. This diverse insect activity underlines the need for a nuanced understanding of how different saproxylic beetle families interact with their woody habitats^19,20^.

In this study, the term habitat association refers specifically to influence of key ecological factors—namely, tree species (Siberian spruce and Siberian pine), decay class (DC1–DC4), forest landscape (north vs. south mixed forest) and their interactions on saproxylic beetle assemblages. Deadwood serves as habitats for numerous beetle species^12,21^. The decomposition of deadwood creates a variety of microhabitats, supporting a succession of insect communities that change over time as the physical and chemical properties of the wood change^22^. Deadwood classification systems have been proposed, reflecting differences in the decomposition timelines and properties of deciduous and coniferous tree species. Such systems not only guide forest management practices but also help predict the succession patterns of beetle communities within decaying wood. Early decay stages are typically dominated by species that exploit fresh wood, whereas later stages attract species specialized in more decomposed material and associated fungal communities, contributing to the gradual breakdown of the wood^23,24^.

Research has focused on how the quality and quantity of dead wood affect the abundance of these specialized organisms, but there is limited research on the impact of habitat on their damage. In addition, Siberian spruce and Siberian pine trees in the Green zone of Ulaanbaatar were specifically chosen for research on saproxylic beetles—because: 1) these tree species play a significant role in the local ecosystem and offer a unique habitat for a variety of insects, 2) they are dominant species in the mixed forests in the surrounding areas, and 3) both tree species are also of cultural and economic importance in the region, making it valuable to understand the factors that affect their health and longevity, including the impact of saproxylic beetles.

Understanding these contexts is crucial for developing sustainable forest management strategies that balance the ecological benefits of deadwood with the need to mitigate insect-related damage. This study focuses on the influence of tree species and decay classes on saproxylic beetle communities, providing insights that inform effective deadwood management practices. To our knowledge, no other published studies have investigated insect damage along the decay gradient or about the mechanisms driving these patterns for the Siberian spruce and Siberian pine. Thus, this study aims to fill the knowledge gap by assessing the impact of habitat association on the abundance, and damage of saproxylic beetle in downed wood from naturally mixed forests within the Green zone of Ulaanbaatar.

Specifically, the study aimed to compare the saproxylic beetle abundance, guild composition and damage index in Siberian spruce and Siberian pine wood across a gradient of decomposition, ranging from recently deceased to thoroughly decomposed in two forest landscape to address the following questions: 1) How does habitat association affect the abundance and damage severity of saproxylic beetle communities? 2) Are beetle abundance and damage severity being highest in the early decay classes as these stages provide optimal conditions for cambium consumers and wood borers? 3) Which structural characteristics of trees are most strongly correlated with beetle damage and composition? and 4) What species of beetles are most damaging to the Siberian spruce and pine during the early classes of drying? Ultimately, this study contributes to the development of sustainable forest management strategies aimed at minimizing economic losses from beetle infestations while maintaining biodiversity in forest ecosystems.

## Material and methods

### Study area

**Fig. 1:**
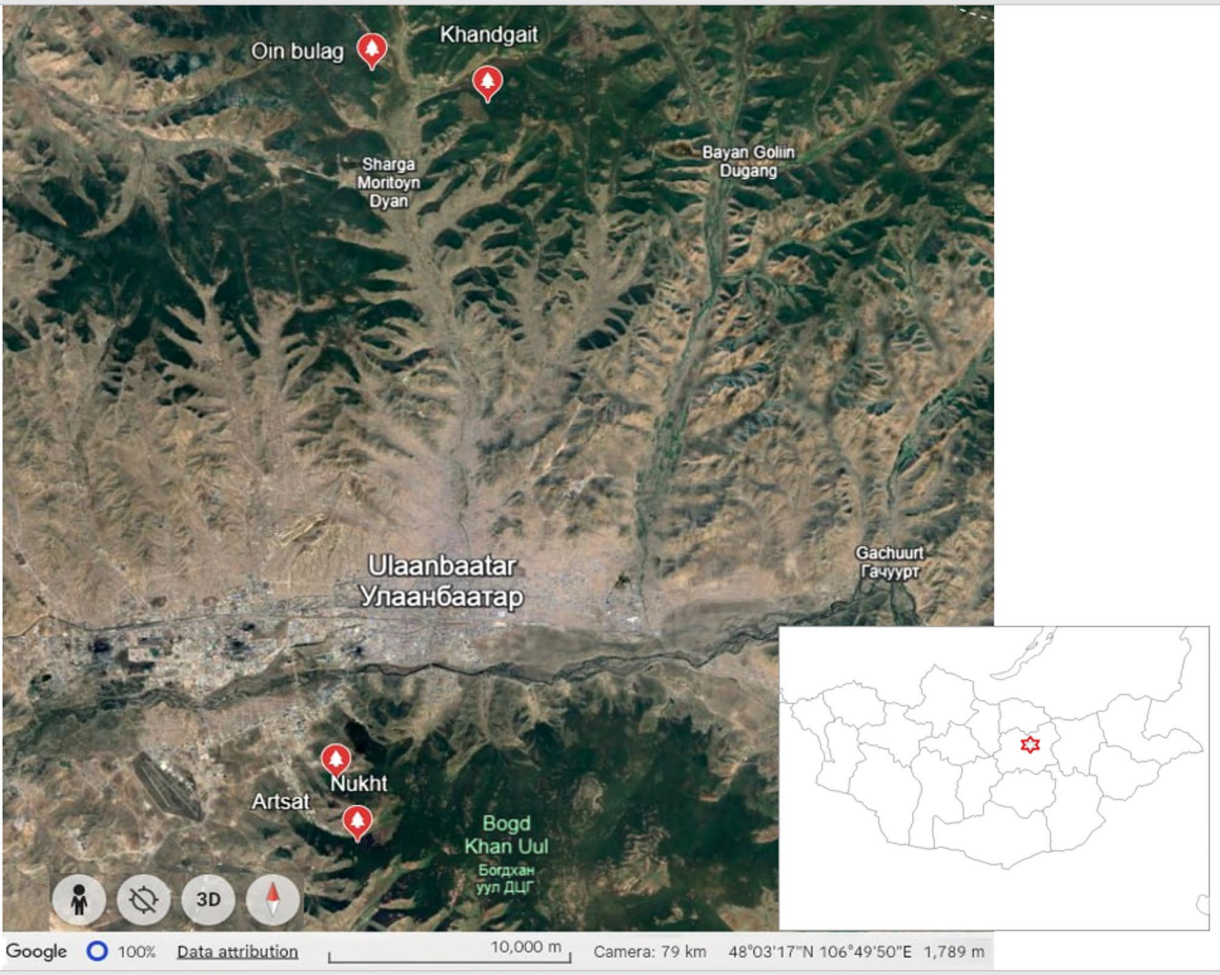
Location of study sites in the Green Zone of Ulaanbaatar, Mongolia. The map highlights Ulaanbaatar, the capital and largest city of Mongolia, in the central part. Red markers indicate the research locations, including Oin Bulag, Khandgait in the north, Artsat, and Nukht in the south part. Satellite imagery is from Google Earth Pro (v7.3.6; https://www.google.com/earth/), freely available for academic and non-commercial use. The map was created and annotated by the authors.

The research covered two mixed forests located in the northern and southern regions of the Green zone of Ulaanbaatar, namely the Bogd Khan Mountain Strictly Protected Area (Nukht N47°49´24˝; E106°51´48˝, Artsat N47°50´53˝; E106°51´13˝) and the Camping area (Khandgait N47°58´42.8˝; E107°25´30˝, Oinbulag N47°45´51˝; E107°07´51˝) (Fig. 1). These two forest landscape sites were selected because of their high diversity, with dark taiga species such as Siberian spruce (*P. obovata*) and Siberian pine (*P.sibirica*), as well as a mix of Scots pine (*Pinus sylvestris* L), Siberian larch (*Larix sibirica* Ledeb), and Asian white birch (*Betula platyphylla* Sukaczev)^25^. Furthermore, Bogd Khan Mountain is a protected reserve, while the forest near Camping area is affected by human recreation.

According to the forest-vegetation zone classification of Mongolia, the study area is located within the Tuul Barkh circle in the Western Khentei Mountain Region^26^. The Bogd Khan Mountain site is approximately 13–20 km south of Ulaanbaatar, with altitudes ranging from 1200 to 1500 m. The northern site, situated in a camping area, is about 30–35 km from Bogd Khan Mountain, at altitudes of 1400–1985 m above sea level.

The climate of the region is influenced by the Asiatic anticyclone during winter, which typically centers around southwest of Lake Baikal, resulting in dry and cold conditions^27^. The temperature in Ulaanbaatar has undergone notable changes over time. Presently, July, the warmest month, averages between + 25 and + 30 °C, whereas temperatures in July were significantly higher, reaching + 35 to + 38 °C. In contrast, January, the coldest month, now averages −24 to −28 °C, but in the past it was much colder, ranging from −30 to −35 °C^28^.

The vegetation includes a shrub layer dominated by species such as *Spiraea media* Schmidt, *Rosa acicularis* Lindl, and *Rubus idaeus* L. The herb layer is characterized by *Fragaria orientalis* Losinsk, *Bisorta alopecuroides* (Kar. & Kir.) Soják, *Bromus inermis* Leyss, *Galium boreale* L, and *Sanguisorba officinalis* L^29^.

### Downed wood samples and decay classification

In the summer of 2020, a total of 80 naturally downed or wind-thrown trees—40 Siberian spruce and 40 Siberian pine—were selected from two forest types (Bogd Khan Mountain and Camping Area) to investigate saproxylic beetle communities. These trees ranged in diameter from 25 to 39 cm and in length from 15 to 21 m. Each species was represented equally in both forest types, and five replicate trees were sampled for each of the four decay classes, totaling 80 samples (2 forest types × 2 species × 4 decay classes × 5 replicates). The rationale for selecting decay classes was based on their ecological relevance to saproxylic beetle succession and decomposition processes in coniferous forests^20^. Trees were systematically assigned to four decay classes (DC1–DC4) based on visual indicators of decomposition, including bark integrity, wood hardness, color, presence of needles, twigs, and branches, as well as signs of fungal colonization and insect activity. The decay classification framework was adapted from^20^, with modifications tailored to conifer species following^12,20,30,31^ (Table 1). Specifically, Harmon’s Classes IV and V were merged into a single advanced decay class (DC4). Additionally, the surface area of fallen tree and branches covered by lichens and mosses was visually estimated on a percentage scale (0–100%)^20^ (Table 1).

**Table 1:** The classification scheme for fallen coarse woody debris (CWD) across four decay classes (DC), each representing progressive stages of decomposition.

Decay Class	Visual Characteristics	Conifer Needles	Twigs and Branches	Bark Cover	Lichens and Mosses
DC 1	Recently downed, minimal decay visible	Present and attached, indicating recent death	Small twigs and branches intact	80–100% retained, tightly adhered to wood	Minimal or absent, little to no colonization
DC 2	Early signs of decay with slight wood softening	Mostly shed; a few needles on larger branches	Smaller twigs beginning to break off, larger branches remain	50–80% retained, peeling or sloughing off in patches	Limited, covering 10% of surface in shaded areas
DC 3	Moderate decay, wood softening, visible fungal colonization	Absent, contributing to forest litter	Most twigs lost; only fragile larger branches remain	20–50% remaining, heavily peeling or flaking off	Moderate, 10–50% coverage, mainly in damp areas
DC 4	Advanced decay with substantial structural breakdown	Absent; fully integrated into forest litter	No small twigs; larger branches decomposed or detached	Less than 20% remains, loose or fully absent	High, 50–100% coverage, extensive colonization

Decay class assignment was based on standardized visual indicators adapted from^20^ and other relevant literature. Key characteristics included bark integrity, twig and branch retention, presence of conifer needles, and epiphytic colonization (lichens and mosses). To ensure consistency with ecological conditions in boreal coniferous forests, we combined Harmon’s Classes IV and V into DC4. All assessments were conducted by the same trained observer, and classification criteria were cross-validated through repeated observations of a subset of logs. This visual approach provides a practical and repeatable method for field-based decay classification in coniferous forests.

No plant specimens were collected or handled during this study. Vegetation information were obtained and cited from previously published sources, specifically Bazarragchaa et al.^29^. Naturally fallen Siberian pine and Siberian spruces trees were identified by the authors and used for this study. The trees are common species, therefore, no formal description or herbarium collection was made. This work complied with institutional, national, and international guidelines and legislation.

### Insect sampling

**Fig. 2:**
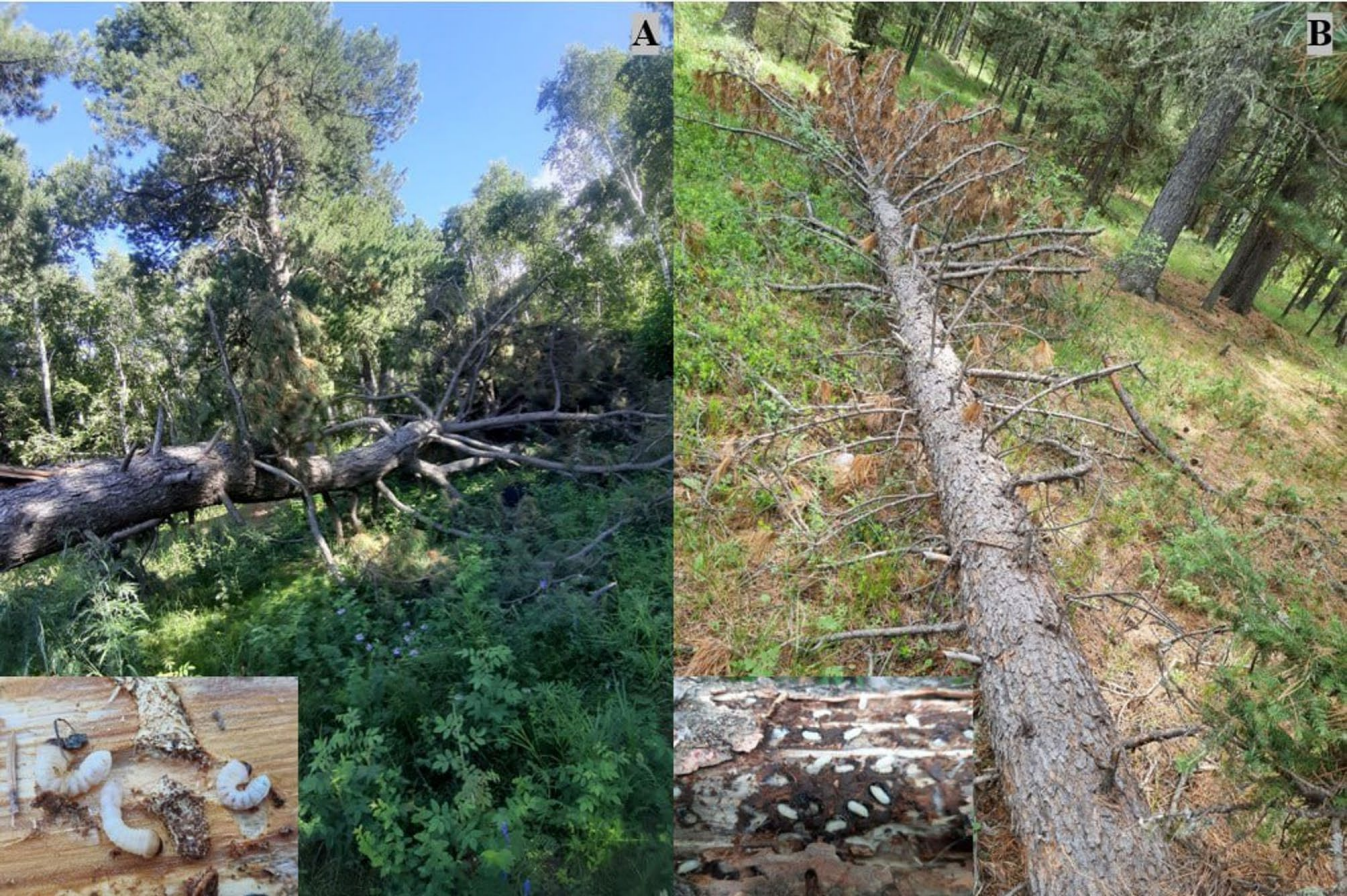
Comparison of saproxylic beetle colonization in fallen trees. (**A**) A fallen Siberian pine in an early decay class and larvae of wood-boring beetles developing under the bark. (**B**) A fallen Siberian spruce in a early decay class and bark beetle galleries in the bark.

Samples were taken from the top, middle, and down sections of fallen Siberian spruce and pine at various decay classes using a 20 × 20 cm^2^ (4 dm^2^) sectioning method^32^. Specifically, the bark was carefully removed using a specialized knife, and the beetles living underneath were identified or recorded by species, and counted (Fig. 2). Samples were collected between 11:00 AM and 3:00 PM on non-rainy days. Larvae and adult specimens were collected in vials containing 70% ethanol for further analysis. Each container was marked with the collection date and location code. Species identification was performed under a laboratory stereoscopic microscope (Motic Panthera C2, Motic Instruments Inc.) to the species level, when possible. Wood moisture was measured using the MMTK-813 Digital Wood Moisture Meter, and bark integrity was assessed as a percentage (10–100%). The study was conducted on 80 fallen trees, with five replicates for each 16 treatment combinations (2 forest types × 2 tree species × 4 decay classes), from July to September in both 2020 and 2021. Additionally, adult insects were trapped using window traps to confirm species identification of larvae and pupae. To minimize microclimatic bias during the study, both the placement of insect traps and the selection of downed trees were based on a randomized sampling approach. Within each forest type (Bogd Khan Mountain and the Camping Area), fallen trees were selected randomly along transects or within stratified plots, with a minimum spacing of 30–50 m to reduce spatial autocorrelation. Traps were placed in microhabitats with relatively homogeneous conditions—specifically on the same orientation (e.g., east- or west-facing side) of each fallen tree. Also, sampling positions were alternated during collection.

Voucher specimens are stored in the beetle reference collection at the Department of Forest Resource and Forest Protection, Institute of Geoecology and Geography, Ulaanbaatar. Species were categorized into five trophic guilds based on literature: cambium and phloem consumers, wood borers, fungivores, predators and detritivores^33,34^.

### Study design and statistical analysis

Our study followed a 2:2*:*4 factorial design with three key factors: forest type (Bogd Khan Mountain and Camping Area), tree species (Siberian spruce and pine), and decay class (DC1-DC4, fresh to highly decayed fallen tree). To assess the influence of categorical variables on the Bevan damage index, we performed nonparametric tests. Specifically, we used the Kruskal–Wallis test to examine whether the Bevan damage index differed among decay classes, tree species, and landscape locations. The analyses were conducted in Jamovi 2.4.8^35^. We used a three-factor analysis of variance to account for habitat influences to abundance and feeding guild activity. In this model, the dependent variables were the beetle abundance and feeding guild activity**.** Forest type, tree species, and decay class (and their interactions) were treated as fixed effects. We assessed the assumptions of normality, homogeneity of variances, and independence for all response variables (beetle abundance and feeding guild abundances) prior to performing three-factor ANOVA. Data were log(x + 1) transformed to reduce skewness and stabilize variances. Normality was evaluated using Shapiro–Wilk’s test and univariate Q-Q plots. Multivariate normality of residuals was assessed through Mahalanobis’ distance vs. Chi-square Q-Q plots, where most points aligned closely with the expected line, indicate satisfactory model fit. Homogeneity of variances was tested using Levene’s test, which showed no significant deviations after transformation. Given the exploratory nature of the study, no formal correction for multiple comparisons was applied. Instead, we interpreted results cautiously, focusing on patterns supported by ecological theory, consistent trends across variables, and statistical significance at the *p* < 0.05 level. These results were visualized using OriginPro 2023b^36^. To reduce spatial dependence, fallen trees were chosen ≥ 30–50 m apart along pre-defined transects within each landscape. Within transects, individual fallen trees were randomly selected from the set meeting inclusion criteria (species, size range, decay class). Sampling was distributed across two landscape types, limiting within-patch clustering. Although precise GPS coordinates for all logs were not recorded and a formal spatial autocorrelation analysis could not be computed, the spacing and randomization procedures were implemented to minimize short-range spatial autocorrelation.

To quantify the severity of beetle-induced damage in fallen trees, we employed the Bevan Damage Index, a widely used metric in forest entomology and ecological monitoring^14^. The index assumes that higher scores correspond to greater beetle colonization intensity and functional impact on wood integrity. Its simplicity, reproducibility, and ecological relevance make it suitable for field-based assessments of saproxylic insect damage, especially in early detection and monitoring programs. In our study, the index allowed for consistent comparison of damage severity across tree species, decay stages, and forest types. After identifying beetles, to calculate Bevan Damage Index, they were divided into five groups based on their economic damage. The Bevan damage index was calculated based on the damage scale and insect frequency^14^. The insect damage scale ranges from 0 to 4. 0: Predator insects that feed on decay fungi; 1: Insects breeding between bark and wood with minor economic impact; 2: Insects damaging the wood surface and those affecting decaying wood; 3: Insects causing significant wood damage; 4: Insects inflicting extensive wood damage, greatly reducing technical specifications and causing major economic losses (Supplementary Table S3). The Damage Index (DI) is calculated using the formula in Eq. (1):

1 $${\text{Damage Index}} = \frac{{{\text{Insect Frequency}} \times {\text{Damage Scale Value}}}}{{\text{Total Damage Index of the Insect Species}}} \times 100$$

where, Insect Frequency is the number of appearances of the insect species in the region at different times; Damage Scale Value is a value assigned according to the damage type based on previous literature studies; Total Damage Index of the insect species is the sum of all individual damage indices for the insect species.

To explore relationships between saproxylic insect feeding guilds and wood decay traits, we performed a Spearman’s rank correlation analysis. All deadwood traits and beetle abundances were measured at the individual tree level (n = 80). Each fallen tree served as a single unit of analysis for correlation tests. The correlation coefficients were visualized using a heatmap, where the color intensity represents the strength of the correlation between feeding guild activity and wood traits (bark cover, conifer, twig, branch, wood moisture, lichen and mosses).

## Results

### Effects of decay class, tree species, and forest landscape on beetle abundance and damage severity

A total of 1559 saproxylic beetle individuals representing 108 species, 97 genius, 36 families were collected from 80 fallen logs across the two forest sites and tree species. Supplementary Table S3 summarizes the total number of each beetle species per tree and site.

**Fig. 3:**
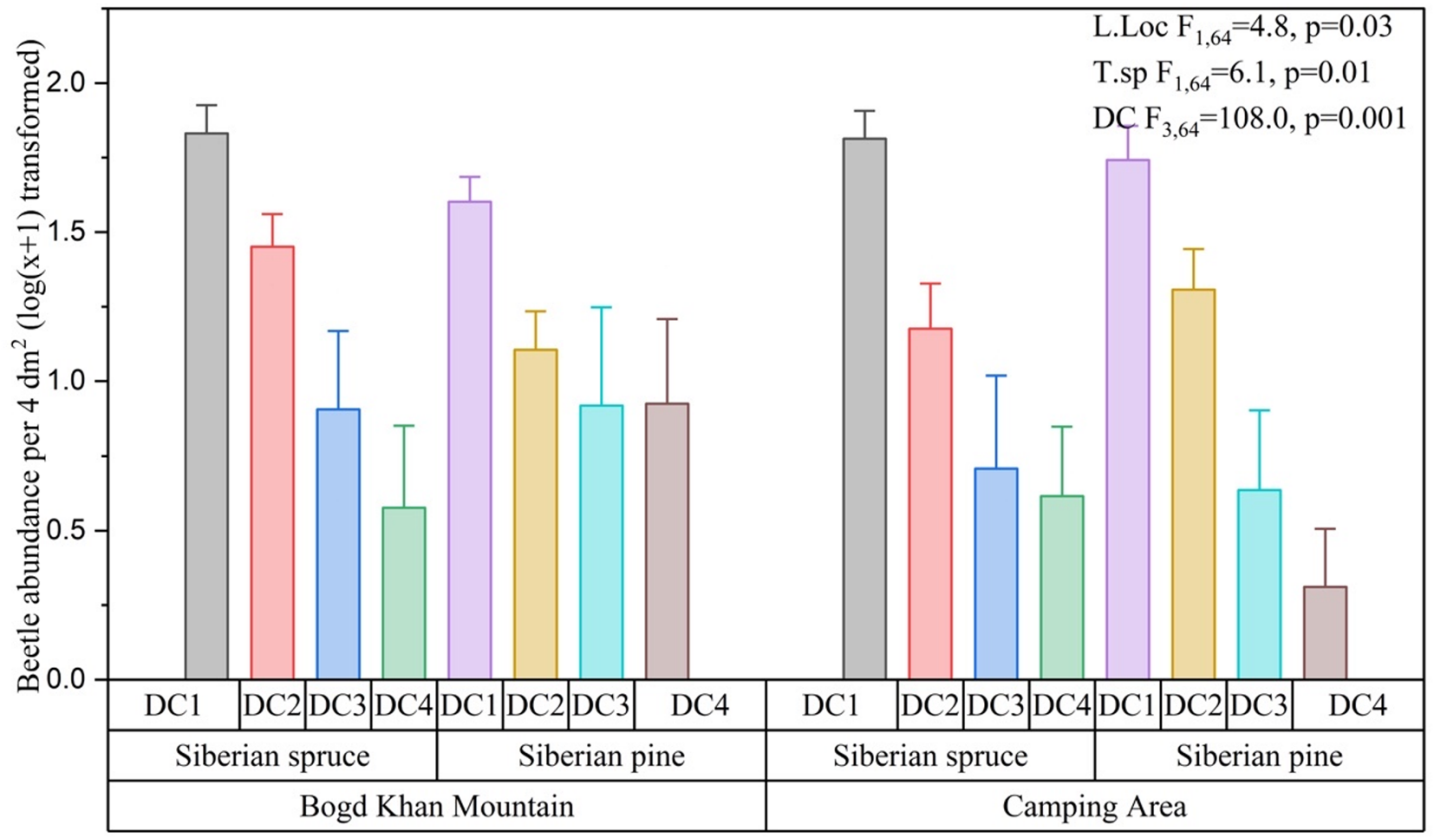
Comparisons of abundance of saproxylic beetles in three habitat characteristics: DC—decay class, T.sp—tree species, L.loc—landscape location (Bogd Khan Mountain-South, Camping forest -north of Green zone of Ulaanbaatar city).

Decay class had a strong effect on beetle abundance (*F*_3,64_ = 119.0, *p* = 0.001) (Fig. 3) with the highest values observed in early decay stages (DC1). In contrast, beetle abundance (*F*₁,₆₄ = 2.0, *p* = 0.1) (Fig. 3) did not differ significantly between Siberian spruce and Siberian pine. For two forest landscapes, no significant effect was observed on both beetle abundance (*F*_1,64_ = 5.6, *p* = 0.06) (Fig. 3).

The Kruskal–Wallis test revealed a significant effect of decay class on the Bevan damage index (χ^2^ = 63, df = 3, *p* < 0.001). In contrast, no significant differences in the Bevan damage index were found between tree species (χ^2^ = 0.196, df = 1, *p* = 0.658) or between landscape locations (χ^2^ = 0.477, df = 1, *p* = 0.49) (Table 2).

**Table 2:** Nonparametric analysis evaluating the impact of habitat.

Effects	χ^2^	df	*p*
Decay class	63	3	< .001
Tree species	0.196	1	0.658
Landscape location	0.477	1	0.49

L.loc-landscape location: Bogd Khan Mountain—South, Camping forest—north of Green zone of Ulaanbaatar city; DC—decay class: DC1 through 4; T.sp—tree species: Siberian pine and Siberian spruce to Bevan damage index.

Among the interaction terms, there was a significant interaction between landscape location and decay class (*F* = 3.12, *p* = 0.032). Additionally, a highly significant three-way interaction among landscape location, tree species, and decay class was observed (*F* = 6.49,* p* = 0.001). The interaction between tree species and decay class (*F* = 0.68, *p* = 0.57) and between landscape location and tree species (*F* = 0.07, *p* = 0.786) were not significant. Full ANOVA results are presented in Supplementary Table S1.

**Fig. 4:**
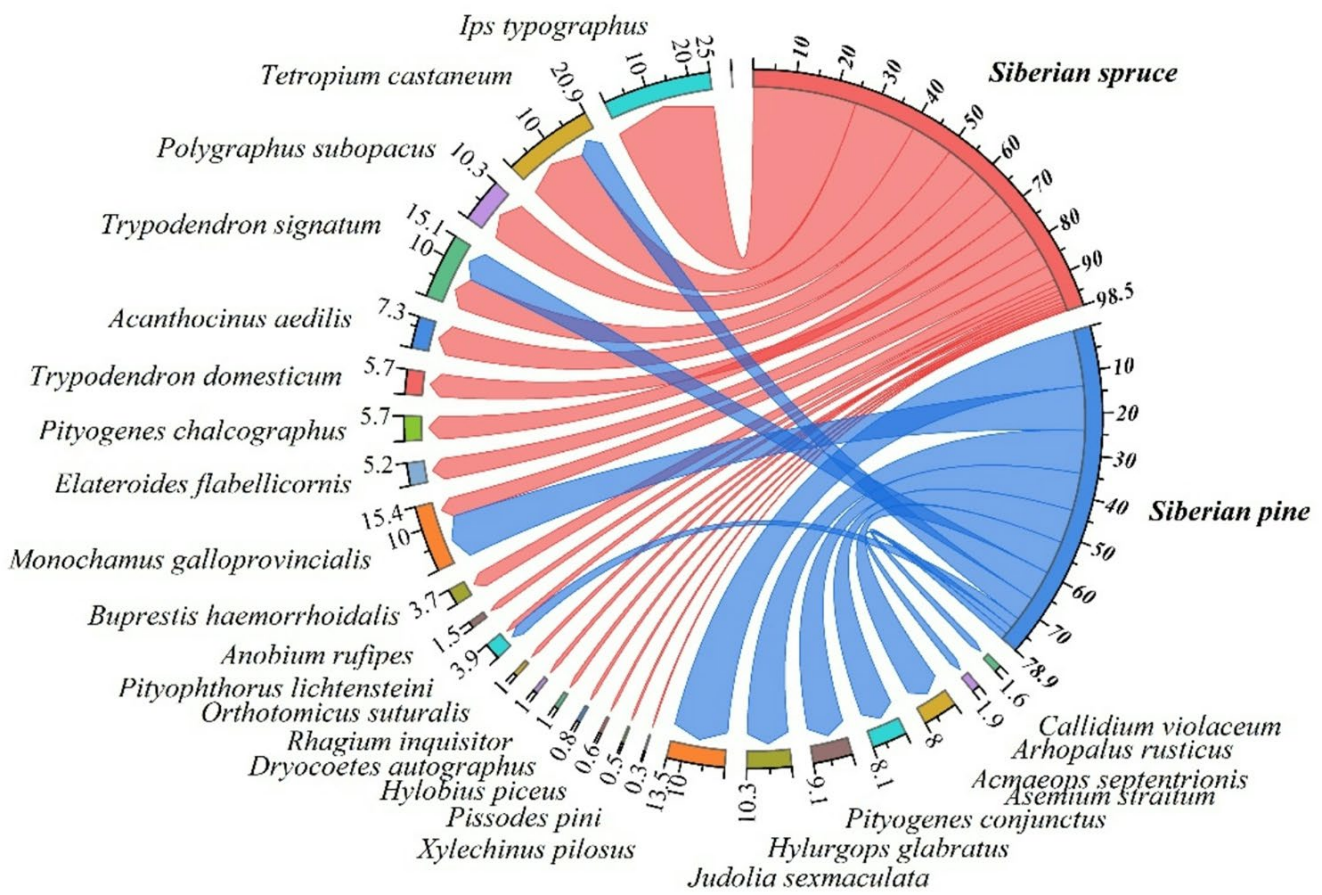
Chord diagram illustrating beetle species distribution and associated damage severity (Bevan Damage Index, BDI) on two tree species (Siberian spruce and Siberian pine) at decay class 1 (DC1). The thickness of each chord represents the relative contribution of each beetle species to the total damage on a given host tree. Red chords indicate damage to Siberian spruce, while blue chords indicate damage to Siberian pine. Numbers adjacent to each beetle species denote its BDI contribution (%) on each host. Significant differences in host preference (*p* < 0.05) were determined based on species-specific abundance data. *I. typographus* and *T. castaneum* showed a pronounced preference for spruce, whereas *J. sexmaculata* and *P. conjunctus* were more associated with pine.

The chord diagram in Fig. 4 illustrates the damage severity of saproxylic insects on Siberian spruce and Siberian pine trees at decay class DC1. Among the species, *I. typographus* (24.7%), *T. castaneum* (15.1%), *Polygraphus subopacus* Thoms (14%), and *Pityogenes chalcographus* L (10%) exhibited the highest levels of damage on Siberian spruce, as shown by the red connections to this tree species, indicating a clear preference for spruce over pine. Conversely, *M. galloprovincialis* (9.8%), *J. sexmaculata* (13.3%), and *P. conjunctus* (10.6%) displayed a stronger association with Siberian pine, with blue connections highlighting their affinity for this host. In contrast, *Melandrya dubia* Schall and *Oedecnema gebleri* Ganglb were the least damaging, with minimal impact (0–1%), represented by their faint and sparse connections to both tree species (Fig. 4). This visualization reveals the host preferences and ecological interactions of saproxylic beetles in early decay class.

### Feeding guild responses to decay class, tree species, and forest landscape

**Fig. 5:**
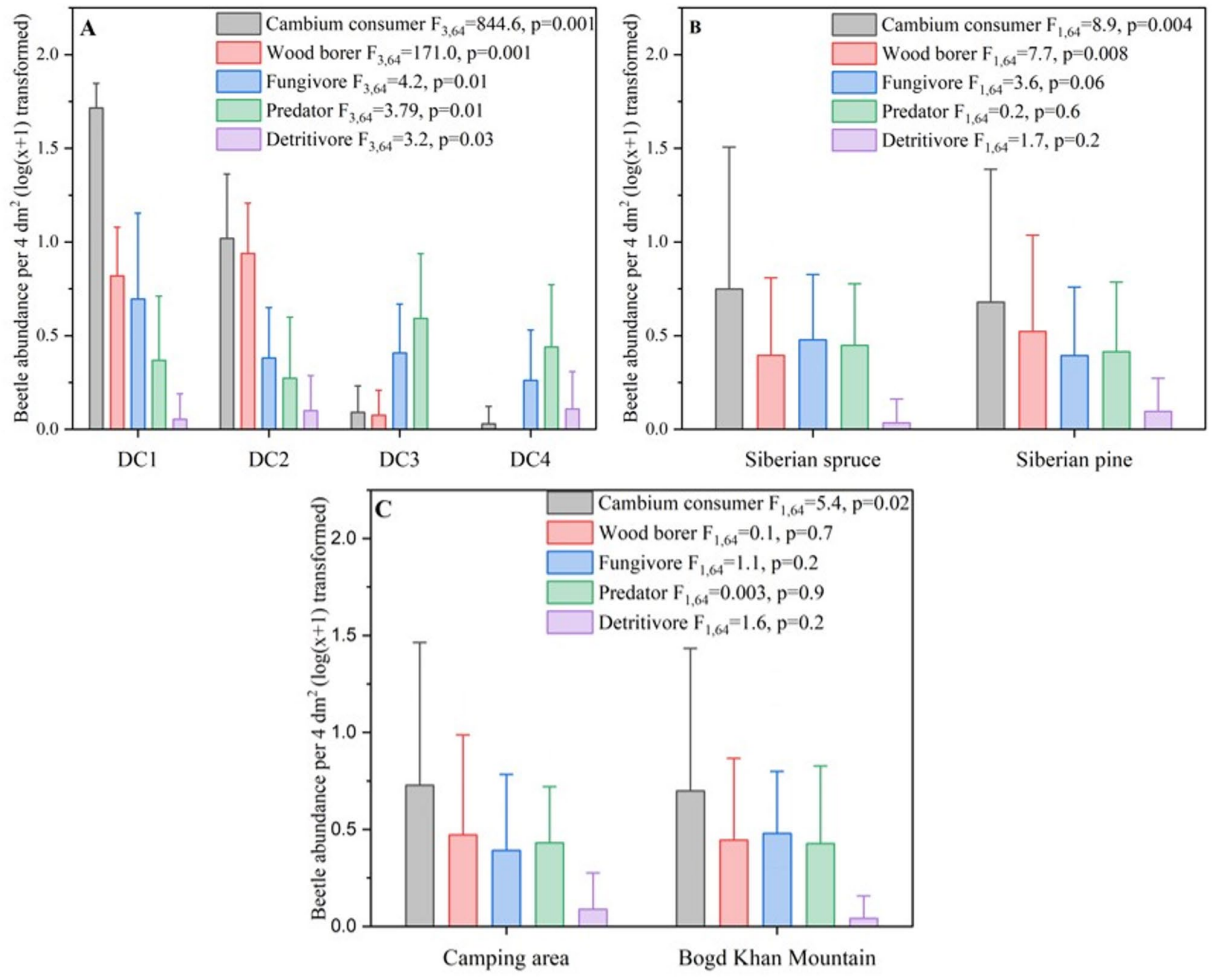
Effect of decay class, tree species and forest location on feeding guild activities (cambium consumer, wood borer, fungivore, predator and detritivore) of forest saproxylic beetles of Green zone of Ulaanbaatar city, Mongolia.

Decay class had a greater influence on the abundance of all functional feeding guilds. Cambium consumers were most abundant in early decay particularly in DC1 (*F*_3,64_ = 844.6, *p* = 0.001), with their numbers decreasing significantly in later decay classes. Wood borers, on the other hand, peaked in DC2 and DC3 (*F*_3,64_ = 171.0, *p* = 0.001), while fungivores and detritivores were more abundant in intermediate and later decay classes, respectively (Fig. 5a).

Significant differences in guild abundance were observed between Siberian spruce and Siberian pine. Cambium consumers were significantly more abundant in Siberian spruce (*F*_1,64_ = 8.9, *p* = 0.004), as were wood borers (*F*_1,64_ = 7.7, *p* = 0.008). No significant differences were found for fungivores (*F*_1,64_ = 3.6, *p* = 0.06), predators (*F*_1,64_ = 0.2, *p* = 0.6), or detritivores (*F*_1,64_ = 1.7, *p* = 0.2)(Fig. 5b).

The abundance of saproxylic feeding guilds varied between the two forest landscapes, with significant differences observed for wood cambium consumer. Cambium consumer were more abundant in the Camping area compared to Bogd Khan Mountain (*F*_1,64_ = 5.4, *p* = 0.02), while no significant differences were found for wood borer (*F*_1,64_ = 0.1, *p* = 0.7), fungivores (*F*_1,64_ = 1.1, *p* = 0.2), predators (*F*_1,64_ = 0.03, *p* = 0.9), or detritivores (*F*_1,64_ = 1.6, *p* = 0.2)(Fig. 5c).

Significant interactions were observed for landscape location × tree species in all guilds except fungivores and detritivores. Three-way interactions (landscape location × tree species × decay class) were significant for cambium consumers (*F*₃,₆₄ = 40.64, *p* = 0.001), wood borers (*F*₃,₆₄ = 7.04, *p* = 0.001), fungivores (*F*₃,₆₄ = 6.43, *p* = 0.001), and detritivores (*F*₃,₆₄ = 6.35,* p* = 0.001), but not for predators (*p* = 0.267) (Supplementary Table S2). Residual variances were low for cambium consumers (0.01) and wood borers (0.02), indicating stable model performance.

### Influence of tree structural characteristics on saproxylic beetle communities and wood damage severity

**Fig. 6:**
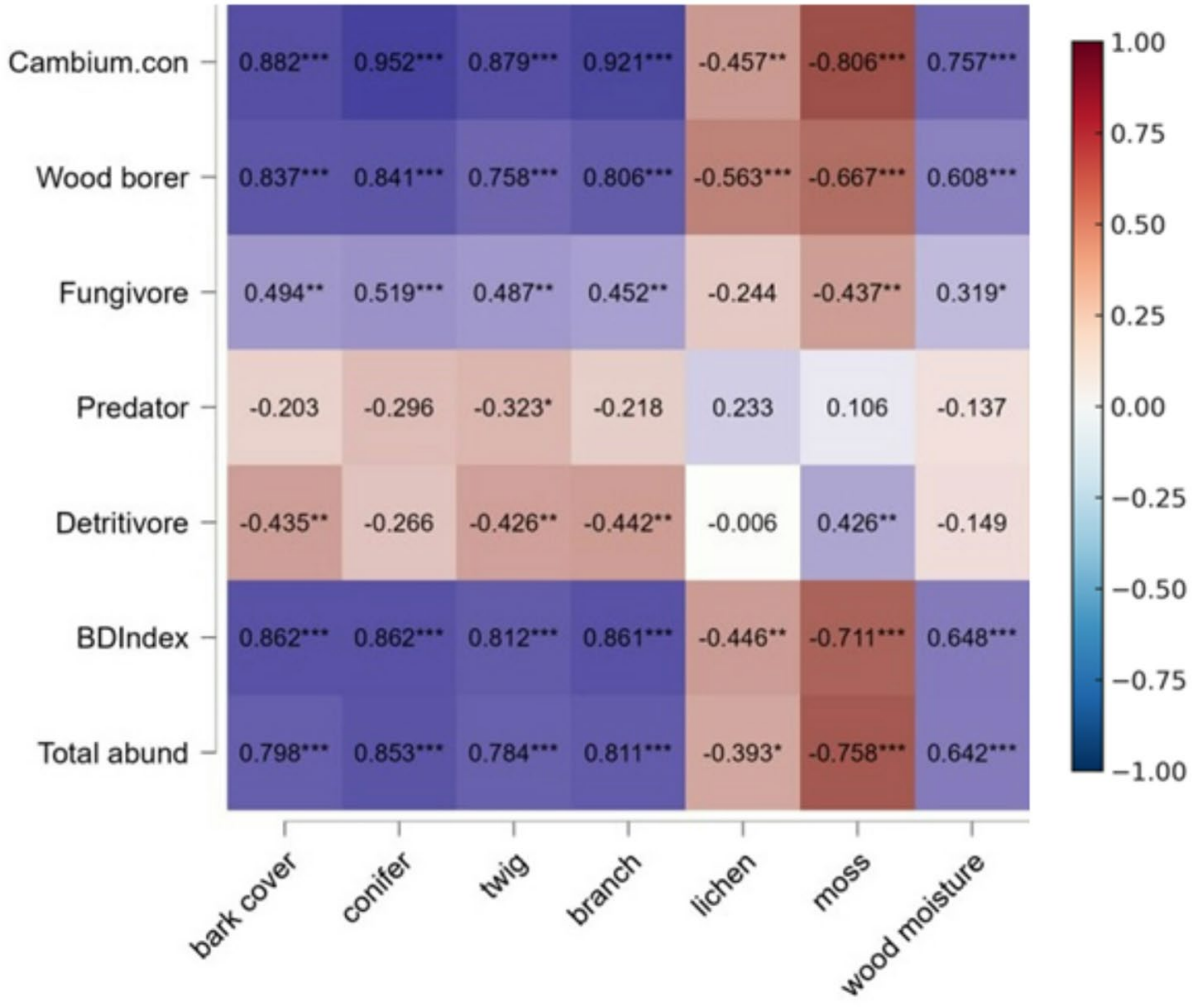
Spearman’s rank correlation matrix heatmap showing the relationship between tree characteristics (horizontal axis) and beetle functional guilds and damage severity (vertical axis) for the two downed tree species. Purple values indicate statistical significance, with levels denoted by stars: *** *p* < 0.001; ** *p* < 0.01; * *p* < 0.05. Brown-marked areas highlight negative correlations, indicating reduced beetle abundance or damage with increasing tree characteristic values.

This study investigates how structural characteristics of fallen trees influence saproxylic beetle communities and wood damage severity across different decay classes. The results show that specific tree features—particularly bark cover, twig presence, and wood moisture—are crucial in shaping beetle assemblages and determining the extent of damage (Fig. 6).

Bark cover exhibited a strong positive correlation with cambium consumer abundance (*r* = 0.83, *p* < 0.001), suggesting that intact bark serves as a key resource and shelter for these beetles in early decay class. Additionally, cambium consumers showed a high correlation with the Bevan damage index (*r* = 0.90, *p* < 0.001). Similarly, twig presence was positively associated with wood borers (*r* = 0.69, *p* < 0.001)(Fig. 6).

Wood moisture levels were positively correlated with cambium consumers (*r* = 0.61, *p* < 0.001) and wood borers (*r* = 0.60, *p* < 0.001). In contrast, detritivores showed a weak or negative correlation with moisture (*r* = -0.23, *p* = 0.05) (Fig. 6).

The Bevan damage index, representing damage severity, was most strongly associated with bark cover (*r* = 0.75, *p* < 0.001) and twig presence (*r* = 0.89, *p* < 0.001). These findings underscore that structural characteristics of fallen trees significantly impact beetle colonization and wood decomposition, offering valuable insights for forest management strategies aimed at mitigating beetle damage during early decay stages to preserve forest health and biodiversity (Fig. 6).

## Discussion

### Damage severity

We studied damage indices of saproxylic beetle and influence of their habitat associations in the Green zone forest of Ulaanbaatar, Mongolia for the first time. Our investigation shows that species belonging to the Cerambycidae and Curculionidae (subfamily Scolytinae) demonstrated the most elevated damage index values when juxtaposed with the damage index values of other taxonomic families. Results also demonstrated that *I. typographus* and *P. subopacus*, members of the Scolytinae subfamily, received elevated scores on the damage index as a result of their increased prevalence in DC1 and greater reproductive potential on spruce. Siberian spruce, characterized by its softer and less resinous wood, is more susceptible to saproxylic insect infestation due to its higher nutrient availability and the relative ease with which insects can penetrate the bark and phloem tissues^37–40^. In contrast, the denser, more resinous structure of Siberian pine provides greater resistance to beetle colonization, making it less prone to damage^41,42^. In the study region, *I. typographus* typically completes one generation per season. Insects in the subfamily Scolytinae are responsible for major tree deaths, causing damage to the cambium layer between wood^43,44^ and bark and their associated fungi mutually benefit from their association, which may contribute to the death of host trees through mycelial penetration, toxin release, or interactions with preformed and induced defenses^45^. Stadelmann et al. (2013) identified several factors that can trigger outbreaks of *Ips typographus*, including an increased heat sum, the volume of standing Norway spruce (*Picea abies*)^46^, the number of infestation spots from the previous year, and major storm events^47,48^. These elements collectively contribute to the conditions favorable for the proliferation of this pest. The predominance of *I. typographus* in causing damage may be similarly attributed to its status as a major pest of Norway spruce, known for its ability to cause widespread tree mortality and economic losses^37,49,50^ and Jurc et al. (2006) observed that *P. chalcographus*, a significant pest of conifer forests^51^, particularly targets Norway spruce^52,53^. This beetle species undergoes two primary life stages annually and tends to inhabit narrow sections of Siberian spruce trunks with thin, smooth bark^50^. Both species show preferences for certain conditions when attacking windthrown spruces, particularly during the first and second years following a storm^48^. Next, *T. castaneum* for Siberian spruce and *M. galloprovincialis* along with *J. sexmaculata* for Siberian pine (Cerambycidae) were identified as the contributors to the high rate of damage affecting the usability and quality of wood production during the early DC1-DC2.

These species were particularly impactful, inflicting significant technical damage on the wood through their larvae, feed on subcortical tissues, including the inner bark, phloem, and immature xylem and later tunnels through the wood to build pupal chambers^54^. This activity not only compromises the structural integrity of the wood but also diminishes its value and suitability for various commercial uses^14,55,56^ explored that Bevan damage classification system was applied to evaluate damage in log depots, revealing that the highest damage was caused by the Cerambycidae (*Arhopalus rusticus L*) and Buprestidae (*Buprestis dalmatina* Mann) families^14,57^ found in their research that *T. castaneum* L., 1758 was the most numerous insects observed in Norway spruce stumps and found that they are not only responsible for transporting pathogenic fungi, but also cambio-xylophagous insects that colonize the stump^57^.

### Effect of decay class and tree species

As expected, our results demonstrate that both decay class and tree species significantly influenced saproxylic beetle abundance and damage severity. Specifically, early-stage spruce logs (DC1–DC2) exhibited the highest levels of beetle colonization and damage, particularly by cambium consumers and wood borers. These findings are directly supported by our empirical data. However, we interpret the greater susceptibility of Siberian spruce relative to Siberian pine as a trend observed in our dataset, rather than a definitive conclusion. This susceptibility is likely linked to structural traits of spruce, such as thicker bark and higher wood moisture, which create favorable conditions for beetle colonization^37^.

Tree species and decay class have been widely recognized as key determinants of saproxylic insect assemblages across multiple studies. For instance^58^, reported a significant increase in Diptera abundance with wood aging, particularly in later decay stages^58^. However, this trend is more evident in decomposer guilds like Diptera, while our study highlights the peak activity of cambium consumers and wood borers in earlier decay stages^31^. Similarly^10^, emphasized the role of tree-specific characteristics and resource quality in shaping beetle communities, consistent with our findings^10^. Also, our results align with those of^59,60^, who reported species-specific preferences for certain tree types and decay stages, reinforcing the role of tree species as a keystone for forest biodiversity^59,60^. Moreover^61,62^, highlighted the influence of deadwood features and ecological networks on saproxylic beetle diversity^61,62^.

Nevertheless, other studies suggest that these are not the only determining factors^63^ emphasized the importance of deadwood spatial connectivity, suggesting that habitat fragmentation could also shape community composition^59,63^. Pointed to the influence of wildfire and forest management, while Bertheau et al. (2009) observed that beetle colonization in exotic tree species may depend on host specificity and other ecological interactions^61,64^. These perspectives underscore the complex and context-dependent dynamics of beetle colonization.

Deadwood decay stage remains a critical ecological factor shaping not only beetle^61^ but also fungal^17^ and bacterial communities^11^. Our results showed peak beetle activity in DC1 and DC2, aligning with previous findings that early decay stages provide optimal moisture and intact bark—favorable for cambium consumers and borers^59^. As decay advances (DC3–DC4), declining moisture and nutrient levels likely constrain beetle colonization. This reinforces the need for early-stage intervention in deadwood management^59^. Recent research by Zarges et al.^65^ supports this approach, showing that debarking harvesters effectively suppress *Ips typographus* populations while conserving non-target beetles—offering a viable strategy for integrated pest and biodiversity management^66^.

Compared to regional studies—for example^66^, in the Russian Taiga and Wu (2008) in southwestern China—our results similarly highlight the significance of decay stage and host tree characteristics in structuring saproxylic beetle communities^67,68^. However, variation in dominant taxa and guild composition across regions likely reflects differences in regional climate, forest structure, and management practices^73^. These spatial and ecological variations emphasize the need to ground forest pest‑management strategies in local ecological context rather than applying broad generalizations across biomes.

### Feeding functional guilds

Feeding guild structure changed with wood decay, with cambium consumer-phloeophagous beetles dominating the early decay stages but decreasing in abundance in the later stages, where they were gradually replaced by predators, mycetophagous and parasitoid insects. Overall, cambium consumer was higher than other guilds in Siberian spruce (93%) related to DC1 as well as in Siberian pine (65%). This pattern is likely driven by the presence of intact bark and sufficient wood moisture, which together create a suitable microhabitat for feeding and reproduction. Similar results were found by researchers. Savage (2018) recorded 76% of phloeophagous insects in DC1 in pine, while Lee (2018) recorded 81% in the same decay class in white spruce log (*Picea glauca* (Moench) Voss)^69^. The phloeophagous feeding habits of a group of bark beetles, namely *I. typographus* L,1758, *P. subopacus* Thoms, 1865, *P. chalcographus* L.,1761, and *P. conjunctus* Reitt, 1887 indicate that they may be involved in the processes that transfer this stage of coarse wood debris to the next decay class. Our findings indicate that wood borers are more abundant in the Camping area than in Bogd Khan Mountain, which may be explained by microclimatic differences and human disturbance in recreational forests. Increased light exposure, higher temperature variability, and variations in understory vegetation can create favorable microhabitats for wood-boring beetles, whereas other saproxylic feeding guilds appear less sensitive to such landscape-level differences.

Contradictorily, predatory and detritivores insects were observed to be less affected by tree species and decay categories, as their ecological role may be little dependent on the physical properties of the substrate and more on the availability of prey or detritus. Predatory insects may be more responsive to prey abundance, which can be influenced by factors other than tree species and decay class^70^. However, according to^59^, predators made up the largest proportion of the total abundance in the later stages. This various feeding strategies, such as cambium consumers, wood-borers, predators, mycetophagids, and detritivores reflect the diverse ecological niches, feeding strategies, and behaviors of saproxylic insects and trophic interactions within deadwood habitats^11,71^.

### Effect of wood characteristics

Our findings reveal that bark cover and branch size are strongly correlated with the Bevan damage index in Siberian spruce. This suggests that the thicker and more continuous bark of Siberian spruce makes it a more favorable substrate for cambium consumers and wood borers, compared to the thinner bark of Siberian pine. Several studies suggest that the condition of the bark can influence beetle colonization and infestation. For instance^16^, that the colonization of trees by beetles initiated most frequently in the upper thin-barked part of the trunk and in thick branches, suggesting that certain bark characteristics, possibly including its adherence to the wood, are relevant to beetle colonization^16^. Studies have shown that bark traits can significantly influence invertebrate communities in fallen tree, including cambium consumers, at different stages of decay^72^. The decay class of wood affects its structural integrity and nutrient content, which in turn influences the community composition of wood-inhabiting organisms^73^. For instance, the presence of bark can alter wood decay rates, and thus, the habitat suitability for these organisms^74^. Additionally, the decomposition process of bark itself can affect wood decomposition, which is relevant for species that rely on both bark and wood^75^. Furthermore, the decay class can influence the nutrient dynamics and ionic composition of the wood, which are critical for the survival and reproduction of cambium consumers and woodborers^73^. The presence of bark has been shown to significantly affect wood decay rates and insect activity, with bark-covered logs losing more mass and having different insect interactions compared to logs without bark^22^. Wood moisture levels were positively correlated with cambium consumers and wood borers, highlighting that moist conditions support microbial growth and soften wood fibers, thereby facilitating beetle colonization. In contrast, detritivores showed a weak or negative correlation with moisture, reflecting their preference for drier conditions often found in later decay classes. Factors such as bark thickness, integrity, and moisture content influence the microhabitat selection of some insect prey species^76^. These factors can influence the suitability of dead wood for different insect species, affecting their abundance and diversity^63^. Interestingly, while some studies have shown that certain saproxylic insects prefer moist conditions often associated with the presence of bark, which can retain moisture and provide a microhabitat for fungi and other food sources^31^, others have highlighted the importance of sun-exposed coarse woody debris, which may have less bark and potentially lower moisture content, for saproxylic diversity^65,77^. Additionally, the presence of bark may affect the thermal properties of dead wood, influencing the microclimate and the insect communities that it supports.

### Limitations

We acknowledge several limitations of this study. First, the research was geographically confined to boreal forests in the Green Zone of Ulaanbaatar, which may limit generalizability to other forest ecosystems. Second, only two coniferous tree species were examined, and our study spanned a relatively short time frame of two years, which may not capture long-term successional patterns of saproxylic beetles. Third, while our sample size (*n* = 80) allowed for robust comparisons across decay classes and tree species, broader-scale studies are needed to further validate these patterns under varying ecological and climatic conditions. Next, we did not retain precise coordinates for all sampled logs; consequently, we could not apply a formal spatial autocorrelation test (e.g., Moran’s I). Our design (≥ 30–50 m spacing, randomized selection within transects, sampling across two landscapes) was intended to minimize short-range spatial dependence, yet residual autocorrelation cannot be entirely excluded. Given the magnitude and consistency of the decay-class effects, we consider the principal conclusions to be robust.

Lastly, our interpretation focused on pre-specified ecological hypotheses (e.g., the effects of decay class and tree species) while avoiding post-hoc comparisions, since it is common knowledge that conducting multiple comparisons without formal adjustments (e.g., Bonferroni correction) increases the risk of Type I error. However, given the consistency of observed patterns across multiple response variables (e.g., beetle abundance, damage index, and feeding guild structure), we believe that our key findings remain robust and ecologically meaningful. Future studies with larger sample sizes, expanded geographic coverage, and long-term monitoring are recommended to further validate and generalize these results.

## Management and conservation implications

Our study demonstrates that saproxylic beetle damage is most severe in the early decay stages (DC1–DC2), particularly in Siberian spruce. This finding emphasizes the critical importance of decay‐stage‐based management in boreal conifer forests. Early decay stages provide the most suitable conditions for cambium consumers and wood borers due to intact bark, higher wood moisture, and abundant small branches, which collectively create favorable microhabitats for colonization.

To mitigate economic losses, forest managers should prioritize early detection and monitoring of freshly fallen trees during spring (April–May), before beetle colonization begins. Selective utilization of high‐risk logs—especially spruce in DC1—and mechanical debarking of freshly downed trees can substantially reduce available breeding habitat for saproxylic pests such as *Ips typographus* and *Tetropium castaneum*, which are particularly damaging to fallen trees. These interventions allow for the recovery of usable timber while simultaneously lowering the risk of secondary outbreaks.

In contrast, later decay stages (DC3–DC4) are ecologically valuable but pose less economic risk, as they are mainly colonized by non‐target decomposers, fungivores, and detritivores that contribute to nutrient cycling and forest biodiversity. Retaining this deadwood in situ supports ecosystem functions, such as soil enrichment and the conservation of rare saproxylic species.

By applying stage‐specific management strategies, forest managers can achieve a balanced approach: early intervention in high‐risk decay stages to protect timber quality and reduce beetle damage, while conserving later‐stage deadwood for biodiversity benefits. Such integrated management aligns with sustainable forestry goals by safeguarding both forest health and economic interests in boreal conifer ecosystems^65,77^.

## Conclusions

This study emphasizes the critical role of decay class and tree species in influencing saproxylic beetle dynamics in Siberian boreal forests. Our findings show that Siberian spruce may be more susceptible to beetle colonization and damage than Siberian pine, particularly during early decay stages. Notable families such as Curculionidae (subfamily Scolytinae) and Cerambycidae appear to be the primary drivers of damage severity, highlighting the need for targeted decay stage management to mitigate beetle impact.

Effective forest management must balance the ecological value of saproxylic beetles with the potential economic losses they cause. In stands with satisfactory health status, where beetle populations remain at background levels (the so-called “iron reserve”), maintaining deadwood is beneficial for biodiversity and does not pose a serious risk. However, during outbreak stages, where beetle populations increase massively, stricter measures—such as removing infested material—may be required to limit their spread and reduce tree mortality.

Accurate evaluation of saproxylic beetle damage and habitat associations can inform forest management decisions aimed at protecting both timber quality and ecosystem health. Implementing decay-stage-based wood storage strategies—tailored to tree species and structural conditions—can significantly reduce beetle impacts, particularly during the first year after windfall or harvesting. Additionally, incorporating stumps and residual woody debris into forest planning also appears to help balance economic objectives in commercial forests with biodiversity conservation in protected areas.

Our findings support decay-stage-based forest management**,** where early detection, selective utilization, and debarking of freshly fallen logs can mitigate beetle damage during the most vulnerable stages. Retaining later decay stages in place allows biodiversity conservation while minimizing economic losses, offering a balanced approach to sustainable forest management^78^.

Finally, forest management goals differ by landscape type. In protected areas such as parks and reserves, natural processes—including beetle population dynamics—are part of ecosystem functioning and are best monitored rather than interrupted. In contrast, commercial forests may require more proactive intervention to preserve material quality. By integrating ecological knowledge with practical management approaches, it should be possible to maintain forest resilience while safeguarding both biodiversity and economic interests.

## Supplementary Information

Below is the link to the electronic supplementary material.

Supplementary Material 1

## Data Availability

All data supporting the findings of this study are included in the article and its Supplementary Information files.
