# Supplementary material for: Impact of habitat associations on saproxylic beetle assemblages and their damage severity
Source: Sci Rep. 2025 Oct 21;15:36601. doi: 10.1038/s41598-025-20452-5 (PMC12540800; doi:10.1038/s41598-025-20452-5)
Supplement: Supplementary file 1 — Supplementary Material 1 [file 41598_2025_20452_MOESM1_ESM.docx]

**Impact of Habitat Associations on Saproxylic Beetle Assemblages and Their Damage Severity**

***Bat-Amgalan Batchudur ^1^, Nanzaddorj Tsagaantsooj ^1^, Dashzeveg Ganbat^1^, and** ***Bazartseren Boldgiv ^2^**

^1^ Institute of Geoecology and Geography, Mongolian Academy of Sciences, Division of Forest Resource and Forest Protection, Ulaanbaatar, 15170, Mongolia, [batchudurb@mas.ac.mn](mailto:batchudurb@mas.ac.mn), [ntsagaantsooj@yahoo.com](mailto:ntsagaantsooj@yahoo.com), [dgambii6@gmail.com](mailto:dgambii6@gmail.com)

^2^ National University of Mongolia, Department of Biology, Ulaanbaatar, 210646, Mongolia, [boldgiv@num.edu.mn](mailto:boldgiv@num.edu.mn)

*corresponding. [batchudurb@mas.ac.mn](mailto:batchudurb@mas.ac.mn); [boldgiv@num.edu.mn](mailto:boldgiv@num.edu.mn)

**Supplementary Materials**

**Supplementary Table S1.** Results of three-factor ANOVA evaluating the impact of habitat (L.loc-landscape location: Bogd Khan Mountain - South, Camping forest - north of Green zone of Ulaanbaatar city; DC -Decay class; T.sp - Tree species) on beetle abundance. Significant effects (*p* < 0.05) are shown in bold.

| **Effects** | **Dependent Variable** | **df** | **Mean Square** | **F** | **p** |
| --- | --- | --- | --- | --- | --- |
| Landscape location (L.Loc) | Beetle abundance | 1 | 74.113 | 1.09 | 0.301 |
| Tree species (T.sp) | Beetle abundance | 1 | 446.513 | 6.54 | **0.013** |
| Decay class (DC) | Beetle abundance | 3 | 11875.912 | 173.97 | **< .001** |
| L.Loc × T.sp | Beetle abundance | 1 | 159.613 | 2.34 | 0.131 |
| L.Loc × DC | Beetle abundance | 3 | 170.346 | 2.5 | 0.068 |
| T.sp × DC | Beetle abundance | 3 | 455.613 | 6.67 | **< .001** |
| L.Loc × T.sp × DC | Beetle abundance | 3 | 208.913 | 3.06 | **0.034** |
| Residuals | Beetle abundance | 64 | 68.263 |  |  |

**Supplementary Table S2.** Results of three-factor ANOVA evaluating the impact of habitat (L.loc-landscape location: Bogd Khan Mountain - South, Camping forest - north of Green zone of Ulaanbaatar city; DC -Decay class; T.sp - Tree species) on feeding guild activity (cambium consumer, wood borer, fungivore, predator, and detritivore). Significant effects (*p* < 0.05) are shown in bold.

| **Effects** | **Dependent Variable** | **df** | **Mean square** | **F** | **p** |
| --- | --- | --- | --- | --- | --- |
| Landscape location (L.Loc) | Cambium consumer | 1 | 35.1 | 0.74 | 0.393 |
|  | Wood borer | 1 | 40.6 | 5.582 | **0.021** |
|  | Fungivore | 1 | 1.5 | 0.219 | 0.642 |
|  | Predator | 1 | 26.4 | 2.675 | 0.107 |
|  | Detritivore | 1 | 1.0 | 2.746 | 0.102 |
| Tree species (T.sp) | Cambium consumer | 1 | 495.0 | 10.436 | **0.002** |
|  | Wood borer | 1 | 108.1 | 14.861 | **< .001** |
|  | Fungivore | 1 | 5.5 | 0.797 | 0.375 |
|  | Predator | 1 | 7.2 | 0.728 | 0.397 |
|  | Detritivore | 1 | 1.0 | 2.746 | 0.102 |
| Decay class (DC) | Cambium consumer | 3 | 12588.5 | 265.406 | **< .001** |
|  | Wood borer | 3 | 443.2 | 60.923 | **< .001** |
|  | Fungivore | 3 | 125.1 | 18.088 | **< .001** |
|  | Predator | 3 | 38.7 | 3.914 | **0.012** |
|  | Detritivore | 3 | 0.8 | 2.294 | 0.086 |
| L.Loc × T.sp | Cambium consumer | 1 | 812.8 | 17.137 | **< .001** |
|  | Wood borer | 1 | 112.8 | 15.507 | **< .001** |
|  | Fungivore | 1 | 82.0 | 11.854 | **0.001** |
|  | Predator | 1 | 101.2 | 10.24 | **0.002** |
|  | Detritivore | 1 | 0.1 | 0.305 | 0.583 |
| L.Loc × DC | Cambium consumer | 3 | 62.0 | 1.308 | 0.279 |
|  | Wood borer | 3 | 18.9 | 2.604 | **0.059** |
|  | Fungivore | 3 | 4.0 | 0.59 | 0.624 |
|  | Predator | 3 | 26.9 | 2.726 | **0.051** |
|  | Detritivore | 3 | 0.2 | 0.576 | 0.633 |
| T.sp × DC | Cambium consumer | 3 | 343.4 | 7.242 | **< .001** |
|  | Wood borer | 3 | 34.5 | 4.744 | **0.005** |
|  | Fungivore | 3 | 2.7 | 0.397 | 0.756 |
|  | Predator | 3 | 7.9 | 0.799 | 0.499 |
|  | Detritivore | 3 | 0.5 | 1.48 | 0.228 |
| L.Loc × T.sp × DC | Cambium consumer | 3 | 355.5 | 7.496 | **< .001** |
|  | Wood borer | 3 | 82.4 | 11.328 | **< .001** |
|  | Fungivore | 3 | 125.6 | 18.16 | **< .001** |
|  | Predator | 3 | 3.1 | 0.319 | 0.812 |
|  | Detritivore | 3 | 1.6 | 4.463 | **0.007** |
| Residuals | Cambium consumer | 64 | 47.4 |  |  |
|  | Wood borer | 64 | 7.2 |  |  |
|  | Fungivore | 64 | 6.9 |  |  |
|  | Predator | 64 | 9.8 |  |  |
|  | Detritivore | 64 | 0.3 |  |  |

**Supplementary Table S3.** Beetle species collected in fallen tree of forest sites in Green zone of Ulaanbaatar city, Mongolia

| **family** | | ***Beetle species*** | **Feeding** | **Damage scale** | **Camping Area** | | **Bogd khan** | | **decay class** |  |
| --- | --- | --- | --- | --- | --- | --- | --- | --- | --- | --- |
|  |  |  |  |  |  |  |  |  |  |  |
|  |  |  |  |  | **Spruce** | **pine** | **Spruce** | **pine** |  |  |
| *Anobiidae* | | *Anobium rufipes* (Fabricius, 1792) | M,W | 4 | 0 | 0 | 3 | 0 | DC1,2 |  |
| *Anobiidae* | | *Dorcatoma dresdensis* (Herbst, 1792) | W | 4 | 0 | 0 | 4 | 0 | DC3 |  |
| *Boridae* | | *Boros schneideri* (Panzer, 1795) | P | 0 | 8 | 2 | 1 | 1 | DC1,2 |  |
| *Bostrichidae* | | *Stephanopachys substriatus* (Payk., 1800) | P | 4 | 2 | 0 | 0 | 0 | DC2 |  |
| *Buprestidae* | | *Buprestis haemorrhoidalis* (Herbst, 1780) | C,W | 4 | 4 | 0 | 3 | 0 | DC1,2 |  |
| *Cantaridae* | | *Cantharis daurica* (Gebler, 1832) | P | 0 | 2 | 0 | 0 | 2 | DC3,4 |  |
| *Cantaridae* | | *Podabrus alpinus* (Paykull, 1798) | P | 0 | 4 | 0 | 0 | 2 | DC3 |  |
| *Carabidae* | | *Anomotarus* sp. | P | 0 | 0 | 1 | 0 | 0 | DC3 |  |
| *Carabidae* | | *Harminius undulatus* (De Geer, 1774) | P | 0 | 0 | 0 | 1 | 0 | DC2 |  |
| *Carabidae* | | *Bembidion* sp. | P | 0 | 0 | 1 | 2 | 0 | DC1,4 |  |
| *Carabidae* | | *Brachichila* sp. | P | 0 | 0 | 0 | 1 | 0 | DC1 |  |
| *Carabidae* | | *Calathus* sp. | P | 0 | 0 | 0 | 1 | 0 | DC3 |  |
| *Carabidae* | | *Carabus canaliculatus* (Adams, 1812) | P | 0 | 0 | 3 | 0 | 2 | DC3,4 |  |
| *Carabidae* | | *Dromius* sp. | P | 0 | 0 | 1 | 0 | 2 | DC4 |  |
| *Carabidae* | | *Harphalus* sp. | P | 0 | 6 | 0 | 1 | 2 | DC2,3 |  |
| *Carabidae* | | *Pterostichus* sp. | P | 0 | 0 | 2 | 0 | 0 | DC4 |  |
| *Carabidae* | | *Tachyta nana* (Gyllenhal, 1810) | P | 0 | 0 | 0 | 1 | 0 | DC4 |  |
| *Carabidae* | | *Taphoxenus* sp. | P | 0 | 0 | 2 | 0 | 0 | DC4 |  |
| *Cerambycidae* | | *Acanthocinus aedilis*(Linnaeus, 1758) | W,C | 3 | 14 | 0 | 0 | 0 | DC1 |  |
| *Cerambycidae* | | *Acmaeops septentrionis* (Thomson, 1866) | W,C | 3 | 0 | 0 | 1 | 10 | DC1,2 |  |
| *Cerambycidae* | | *Arhopalus rusticus* (Linnaeus, 1758) | W,C | 4 | 0 | 2 | 0 | 1 | DC1 |  |
| *Cerambycidae* | | *Asemium straitum* (Linnaeus, 1758) | W,C | 3 | 0 | 11 | 0 | 0 | DC1 |  |
| *Cerambycidae* | | *Callidium aeneum* (De Geer, 1775) | W,C | 3 | 2 | 0 | 0 | 1 | DC1,4 |  |
| *Cerambycidae* | | *Clytus arientoides* (Linnaeus, 1758) | W,C | 3 | 0 | 1 | 0 | 0 | DC1 |  |
| *Cerambycidae* | | *Exocentrus conjugatofasciatus* (Tsherepanov, 1973) | W,C | 3 | 0 | 0 | 2 | 0 | DC2 |  |
| *Cerambycidae* | | *Judolia sexmaculata* (Linnaeus, 1758) | W,C | 3 | 0 | 62 | 0 | 8 | DC1,2 |  |
| *Cerambycidae* | | *Monochamus galloprovincialis* (Olivier, 1795) | W,C | 4 | 19 | 42 | 9 | 0 | DC1,2 |  |
| *Cerambycidae* | | *Monochamus saltuarius* (Gebler, 1830 ) | W,C | 4 | 12 | 0 | 0 | 10 | DC2 |  |
| *Cerambycidae* | | *Monochamus sutor* (Linnaeus, 1758) | W,C | 4 | 0 | 0 | 0 | 7 | DC2 |  |
| *Cerambycidae* | | *Necydalis major* (Linnaeus, 1758) | W,C | 3 | 0 | 0 | 10 | 0 | DC2 |  |
| *Cerambycidae* | | *Pogonocherus fasciatus* (De Geer in 1775) | W,C | 3 | 0 | 0 | 1 | 0 | DC2 |  |
| *Cerambycidae* | | *Rhagium inquisitor* (Linnaeus, 1758) | W,C | 4 | 10 | 0 | 2 | 1 | DC1 |  |
| *Cerambycidae* | | *Tetropium castaneum* (Linnaeus, 1758) | W,C | 2 | 57 | 4 | 34 | 18 | DC1,2 |  |
| *Cucujidae* | | *Pediacus dermestoides* (Fabricius, 1792) | D | 0 | 2 | 3 | 0 | 1 | DC3,4 |  |
| *Cisidae* | | *Cis boleti* (Scopoli, 1763) | M | 0 | 0 | 4 | 0 | 0 | DC1 |  |
| *Cleiridae* | | *Thanasimus femoralis* (Zetterstedt, 1828) | P | 0 | 0 | 5 | 0 | 4 | DC3 |  |
| *Cleiridae* | | *Thanasimus rufipes*(Brahm, 1797) | P | 0 | 12 | 0 | 0 | 0 | DC1 |  |
| *Cleiridae* | | *Thanasimus substriatus* (Gebler, 1841) | P | 0 | 0 | 0 | 8 | 0 | DC3 |  |
| *Cleiridae* | | *Trichodes ircutensis* (Laxmann, 1770) | P | 0 | 4 | 0 | 0 | 0 | DC2 |  |
| *Coccinelidae* | | *Harmonia axyridis* (Pallas, 1773) | P | 0 | 0 | 0 | 1 | 0 | DC2 |  |
| *Coccinellidae* | | *Anatis ocellata* (Linnaeus,1758) | P | 0 | 2 | 0 | 0 | 0 | DC4 |  |
| *Corylidae* | | *Epuraea* sp. | P | 0 | 0 | 4 | 0 | 0 | DC3 |  |
| *Cryptophagidae* | | *Cryptophagus dorsalis*(Sahlberg, 1819) | P, M | 0 | 0 | 0 | 7 | 0 | DC1,3,4 |  |
| *Curculionidae* | | *Cryptorhynchus lapathi* (Linnaeus, 1758) | C | 2 | 0 | 0 | 0 | 1 | DC2 |  |
| *Curculionidae* | | *Hylobius piceus*(De Geer, 1775) | C | 1 | 3 | 0 | 8 | 0 | DC1 |  |
| *Curculionidae* | | *Pissodes pini*(Linnaeus, 1758) | C | 1 | 21 | 0 | 4 | 0 | DC1,2 |  |
| *Dermestidae* | | *Globicornis rufitarsis* (Panzer, 1796) | M,D | 0 | 0 | 0 | 0 | 1 | DC2 |  |
| *Carabidae* | | *Agonum* sp. | P | 0 | 1 | 0 | 2 | 0 | DC3 |  |
| *Elateridae* | | *Ampedus nigror* (Reitter, 1896) | P,D | 0 | 2 | 0 | 0 | 2 | DC4 |  |
| *Elateridae* | | *Athous* sp. | P, D | 0 | 1 | 0 | 0 | 2 | DC2 |  |
| *Elateridae* | | *Danosoma fasciatus* (Linnaeus, *1758)* | P | 0 | 4 | 0 | 0 | 0 | DC2 |  |
| *Elateridae* | | *Selatsomus melanchoicus* (Fabricius, 1798) | P,D | 0 | 1 | 0 | 0 | 0 | DC4 |  |
| *Erotylidae* | | *Triplax collaris* (Schaller, 1783) | M | 0 | 4 | 0 | 9 | 3 | DC2,3,4 |  |
| *Leiodidae* | | *Anisotoma glabra*(Fabricius, 1787) | M | 0 | 0 | 0 | 4 | 3 | DC2,3,4 |  |
| *Lucanidae* | | *Sinodendron cylindricum* (Linnaeus, 1758) | M,C | 2 | 1 | 0 | 0 | 0 | DC3 |  |
| *Lycidae* | | *Clyster* sp. | M | 0 | 0 | 0 | 0 | 2 | DC4 |  |
| *Lycidae* | | *Platycis cosnardi* (Chevrolat, 1839) | M | 0 | 0 | 10 | 0 | 0 | DC2 |  |
| *Lymexylidae* | | *Elateroides flabellicornis* (Schneider, 1791) | W | 1 | 13 | 0 | 10 | 0 | DC2 |  |
| *Melandrydae* | | *Melandria dubia* (Schaller, 1783) | M | 0 | 0 | 2 | 0 | 3 | DC2 |  |
| *Melandryidae* | | *Serropalpus barbatus*(Schaller, 1783) | M,C | 3 | 8 | 0 | 3 | 0 | DC2,3 |  |
| *Melyridae* | | *Epuraea* sp. | M | 0 | 10 | 0 | 0 | 4 | DC3 |  |
| *Monotomidae* | | *Rhizophagus* sp. | P, M | 0 | 1 | 0 | 0 | 2 | DC1,2,4 |  |
| *Mycetophagidae* | | *Mycetophagus quadripustulatus* (Linnaeus, 1761) | M | 0 | 0 | 0 | 1 | 0 | DC4 |  |
| *Nitidulidae* | | *Epuraea longula* (Erichson, 1845) | M, D | 0 | 0 | 0 | 0 | 4 | DC4 |  |
| *Nitidulidae* | | *Epuraea neglecta* (Heer, 1841) | P,D | 0 | 1 | 0 | 0 | 0 | DC3 |  |
| *Nitidulidae* | | *Epuraea oblonga* (Herbst, 1793) | M, D | 0 | 3 | 0 | 2 | 1 | DC1,2 |  |
| *Nitidulidae* | | *Glischrochilus quadriguttatus* (Fabricius, 1777) | M,P | 0 | 0 | 0 | 0 | 1 | DC2 |  |
| *Nitidulidae* | | *Pityophagus* sp. | M,P | 0 | 1 | 0 | 0 | 0 | DC2 |  |
| *Ptinidae* | | *Ernobius explanatus* (Mannerheim, 1843) | M | 4 | 1 | 0 | 0 | 2 | DC2,3 |  |
| *Pyrochroidae* | | *Pytho depressus* (Linnaeus, 1767) | M | 0 | 0 | 1 | 0 | 0 | DC2 |  |
| *Salpingidae* | | *Phloeodroma* sp. | M | 0 | 0 | 1 | 0 | 0 | DC2 |  |
| *Scolytidae* | | *Dryocoetes autographus* (Eichhoff, 1864) | C | 1 | 8 | 0 | 16 | 0 | DC1,2 |  |
| *Scolytidae* | | *Hylastes opacus* (Erichson, 1836) | C | 1 | 3 | 0 | 0 | 0 | DC1 |  |
| *Scolytidae* | | *Hylurgops glabratus* (Zetterstedt, 1828) | C | 1 | 0 | 37 | 0 | 2 | DC1 |  |
| *Scolytidae* | | *Ips typographus* (Linnaeus, 1758) | C | 1 | 131 | 0 | 13 | 6 | DC1,2 |  |
| *Scolytidae* | | *Orthotomicus suturalis* (Gyllenhal, 1827) | C | 1 | 6 | 0 | 0 | 0 | DC1 |  |
| *Scolytidae* | | *Pityogenes chalcographus* (Linnaeus, 1758) | C | 1 | 50 | 0 | 14 | 0 | DC1 |  |
| *Scolytidae* | | *Pityogenes conjunctus* (Reitter, 1887) | C | 1 | 0 | 41 | 0 | 47 | DC1 |  |
| *Scolytidae* | | *Pityophthorus lichtensteini*(Eichhoff, 1864) | C | 1 | 0 | 0 | 7 | 10 | DC1 |  |
| *Scolytidae* | | *Polygraphus subopacus*(Thomson, 1871) | C | 1 | 58 | 0 | 54 | 0 | DC1 |  |
| *Scolytidae* | | *Trypodendron domesticum*(Linnaeus, 1758) | C | 1 | 33 | 0 | 160 | 0 | DC1 |  |
| *Scolytidae* | | *Trypodendron signatum*(Fabricius, 1787) | M,C | 1 | 0 | 40 | 30 | 0 | DC1,2 |  |
| *Scolytidae* | | *Xylechinus pilosus*(Ratzeburg, 1837) | C | 1 | 0 | 0 | 2 | 0 | DC1 |  |
| *Scraptiidae* | | *Anaspis* sp. | P | 0 | 0 | 0 | 0 | 1 | DC2 |  |
| *Scraptiidae* | | *Orhesia* sp. | P | 0 | 0 | 0 | 2 | 0 | DC1,2 |  |
| *Staphylinidae* | | *Epuraea terminalis* (Mannerheim, 1843) | M, D | 0 | 0 | 4 | 0 | 6 | DC1 |  |
| *Staphylinidae* | | *Epurea longula* (Erichson, 1845) | M, D | 0 | 0 | 0 | 1 | 0 | DC1 |  |
| *Staphylinidae* | | *Lordithon bicolor* (Gravenhorst, 1806) | P | 0 | 0 | 2 | 2 | 4 | DC3,4 |  |
| *Staphylinidae* | | *Nudobius* sp. | P | 0 | 2 | 0 | 0 | 1 | DC1,4 |  |
| *Staphylinidae* | | *Olisthaerus* sp. | M | 0 | 0 | 0 | 1 | 0 | DC1 |  |
| *Staphylinidae* | | *Phloeodroma* sp. | P | 0 | 0 | 0 | 0 | 2 | DC2 |  |
| *Staphylinidae* | | *Phloeonomus lapponicus* (Zetterstedt, 1838) | P | 0 | 0 | 1 | 0 | 2 | DC3 |  |
| *Staphylinidae* | | *Phloeopora* sp. | P | 0 | 0 | 1 | 0 | 4 | DC3,4 |  |
| *Staphylinidae* | | *Phloeostiba* sp. | P | 0 | 0 | 0 | 0 | 4 | DC1 |  |
| *Staphylinidae* | | *Placusa depressa*(Mäklin, 1845) | M,P | 0 | 3 | 4 | 0 | 5 | DC1,3 |  |
| *Staphylinidae* | | *Placusa complanata* (Erichson, 1839) | M,P | 0 | 0 | 0 | 0 | 3 | DC3 |  |
| *Staphylinidae* | | *Plegaderus vulneratus* (Panzer, 1797) | P, M | 0 | 0 | 2 | 2 | 4 | DC1,3,4 |  |
| *Staphylinidae* | | *Quedius fulvicollis* (Stephens, 1833) | P | 0 | 0 | 0 | 0 | 4 | DC3 |  |
| *Staphylinidae* | | *Quedius* sp. | P | 0 | 1 | 1 | 3 | 6 | DC1,3,4 |  |
| *Staphylinidae* | | *Staphylinidae sp1* | P | 0 | 0 | 0 | 0 | 32 | DC3,4 |  |
| *Staphylinidae* | | *Staphylinus dauricus* (Mannerheim, 1830) | P | 0 | 8 | 0 | 0 | 22 | DC2,3 |  |
| *Cleridae* | | *Thanasimus substriatus*(Gebler, 1841) | P | 0 | 4 | 0 | 0 | 0 | DC1,3 |  |
| *Tenebrionidae* | | *Bolitophagus reticulatus* (Linnaeus, 1767) | M | 0 | 0 | 0 | 1 | 0 | DC3 |  |
| *Tenebrionidae* | | *Corticeus* sp. | P | 0 | 0 | 1 | 0 | 0 | DC3 |  |
| *Tenebrionidae* | | *Hypophloeus linearis* (Fabricius 1790) | M | 0 | 1 | 0 | 0 | 1 | DC2 |  |
| *Tenebroinidae* | | *Mycetochara humeralis* (Fabricius, 1787) | P | 0 | 0 | 2 | 0 | 0 | DC3 |  |
| *Tenebroinidae* | | *Prionychus melanarius* (Germar, 1813) | P | 0 | 0 | 0 | 0 | 1 | DC2 |  |
| *Trogossitidae* | | *Ostoma ferruginea* (Linnaeus, 1758*)* | M,D | 0 | 0 | 1 | 0 | 0 | DC2 |  |
|  |  |  |  |  |  |  |  |  |  |  |
